# Establishment and Application of an Index System for the Risk of Drug Shortages in China: Based on Delphi Method and Analytic Hierarchy Process

**DOI:** 10.34172/ijhpm.2022.6360

**Published:** 2022-03-16

**Authors:** Yin Shi, Shusen Sun, Jing Deng, Shao Liu, Tao Yin, Qilin Peng, Zhicheng Gong, Zihua Cheng, Boting Zhou

**Affiliations:** ^1^Department of Pharmacy, Xiangya Hospital, Central South University, Changsha, China.; ^2^National Clinical Research Center for Geriatric Disorders, Xiangya Hospital, Central South University, Changsha, China.; ^3^Hunan Drug Shortage Surveillance and Early Warning Center, Changsha, China.; ^4^The Hunan Institute of Pharmacy Practice and Clinical Research, Changsha, China.; ^5^Department of Pharmacy Practice, College of Pharmacy and Health Sciences, Western New England University, Springfeld, MA, USA.; ^6^Department of Epidemiology and Health Statistics, XiangYa School of Public Health, Central South University, Changsha, China.; ^7^Department of General Surgery, Xiangya Hospital, Central South University, Changsha, China.

**Keywords:** Drug Shortages, Index System, Delphi Method, Analytic Hierarchy Process, China

## Abstract

**Background:** At present, the avoidance of drug shortages mainly relies on expert experience. This study aimed to establish an evaluation index system for the risk of drug shortages in medical institutions in China and to apply the system to guide the graded management of drugs in short supply.

**Methods:** A two-round Delphi process was conducted to determine the indicators in the index system. The weight value of each indicator was calculated using analytic hierarchy process (AHP) methods. The data of drugs in short supply from January 1 to December 31, 2020 in Hunan province were collected and evaluated using this index system. The evaluation scores, which ranged from 0 to 100, were calculated.

**Results:** A three-level index system with four first-level indicators, 11 second-level indicators, and 36 third-level indicators was constructed by the two rounds of the Delphi process. The expert authority coefficient (*Cr*) of the first and second rounds of consultation were 0.88 and 0.90, respectively. The Kendall’s coefficients of concordance (Kendall’s *W*) for the two rounds of consultation were 0.44 and 0.50, respectively (*P*<.05). For the first-level indicators ‘supply stability,’ ‘causes of shortage,’ ‘medicine availability in medical institution’ and ‘pharmaceutical properties,’ the weight values were 0.3253, 0.2489, 0.2398, and 0.1860, respectively. Based on the risk evaluation score, drugs (dosage strength) at high risk of shortage included sodium thiosulfate (0.64 g), posterior pituitary lobe hormones (1 mL:6 IU), protamine sulfate (5 mL:50 mg), thrombin (500 U), urokinase (10 WU), and rotundine sulfate (2 mL:60 mg).

**Conclusion:** An indexed system for the risk assessment of drug shortages in China was established to guide the graded response to drug shortages in medical institutions and the implementation of differential management strategies to address these shortages.

## Background

**Key Messages**

** Implications for policy makers**

- A hierarchical indexed system including four first-level indicators, 11 second-level indicators, and 36 third-level indicators for the risk assessment of drug shortages in China was established for the first time using a modified Delphi process with analytic hierarchy process (AHP).
- Among all first-level indicators, ‘supply stability’ had the highest weight value, indicating that it had the greatest impact on shortage risk.
- We carried out a risk assessment of drug shortages in Hunan province using the index system and identified the following six definite high-risk drugs (dosage strength): sodium thiosulfate (0.64 g), posterior pituitary lobe hormones (1 mL:6 IU), protamine sulfate (5 mL:50 mg), thrombin (500 U), urokinase (10 WU), and rotundine sulfate (2 mL:60 mg).
- Decision trees for risk-management strategies have been established for the features of drugs at different risk levels (high risk, medium risk, and low risk).
- This study provides an objective and practical tool to guide risk assessment of drug shortages in medical institutions and a scientific basis for the classification and management of drugs with different risk levels.

** Implications for the public**

Drug shortages have become a complex global challenge and have a severe impact on healthcare systems. In the context of limited public health resources, how to scientifically and effectively identify drugs experiencing genuine shortages is critical in addressing drug shortages. At present, the identification and evaluation of drug shortages in China mainly rely on experts’ subjective experience, calling into question whether the results are scientific, reliable, and representative. We established a hierarchical indexed system as an objective and practical tool to guide risk assessment of drug shortages in medical institutions in China for the first time. The risk evaluation scores could provide clues for grading the risk of shortages. We expect that the index system will contribute to the alleviation and solution of drug shortages, and thereby the reduction of disease burden and the establishment of a healthy China.

The World Health Organization (WHO) identified that drug shortages have become a complex global challenge and have a severe impact on the healthcare system.^1-3^ Several factors are contributing to drug shortages, including manufacturing and quality problems, insufficient production capacity, manufacturer business decisions, active pharmaceutical ingredient or raw material shortages or monopolies, and fluctuating drug demand.^4,5^ Moreover, emergency public health crises, such as African swine fever and the coronavirus disease 2019 (COVID-19)^5^ can also pose a challenge to the global drug supply and patients’ access to therapies.

 Drug shortages can affect clinical outcomes, lead to medical errors, endanger the health of patients, and place a heavy burden on healthcare, seriously affecting the safety, efficacy, timeliness, and cost-effectiveness of medication.^6,7^ According to the American Society of Health-System Pharmacists, there were 205 drugs affected by shortages in 2020.^8^ Among these, 129 were new shortages often linked to COVID-19-related drugs such as hydroxychloroquine sulfate.^9^ In China, shortages of 370 drugs were reported in 2020 in Hunan province, according to the Hunan Drug Shortage Surveillance and Early Warning Center.^10^ A study in the United States reported that $209 million in additional costs were associated with the use of more expensive alternatives.^4^ Indeed, our research team found that the shortage of pyridostigmine bromide in Hunan province forced patients to buy drugs out of town and significantly increased indirect costs such as transportation, accommodation, and missed work, all of which accounted for roughly 80% of the total cost, further increased economic burden, and reduced treatment effect and compliance among patients.

 In recent years, China has attached great importance to addressing drug shortages and paying more heed to the early warnings of drug shortage risk than to post-emergency management.^11^ A series of effective measures have been proposed to alleviate and solve drug shortages, such as establishing and improving the three-level (province, city, county) drug use surveillance and early warning network, issuing guidelines on therapeutic alternatives, implementing a management list of drugs in shortage, and providing policy support to the list of drugs such as (1) manufacturers can set their prices for drugs in shortage, (2) medical institutions can purchase drugs in shortage offline without using provincial procurement platforms, and (3) the distribution of drugs in shortage is exempt from the two invoice policy.^12-14^ Several provinces, such as Yunnan, Ningxia, Shanxi, Guizhou, Guangxi, and Hainan have successively released a list of drugs in shortage since 2019.^15-20^ Recently, a national list of drug shortages and a national list monitoring clinically essential drugs vulnerable to shortages were also released for the first time.^21^

 There are hundreds of drugs experiencing varying degrees of shortage each year. This shortage varies greatly across regions and levels of healthcare. In the context of limited public health resources, how to scientifically and effectively identify and evaluate drugs experiencing genuine shortages is critical to solving drug shortages. However, as the government has not yet issued relevant methodological guidance, the evaluation of drug shortages in China mainly relies on experts’ subjective experience, calling into question whether the results are scientific, reliable, and representative. Therefore, it is necessary to establish a quantifiable evaluation index for assessing the risk of drug shortages.

 Using a modified Delphi method and the analytic hierarchy process (AHP), this study established an index system for evaluating the risk of drug shortages in China and applied it to guide the graded management of drugs in shortage in medical institutions.

## Methods

**Figure 1:**
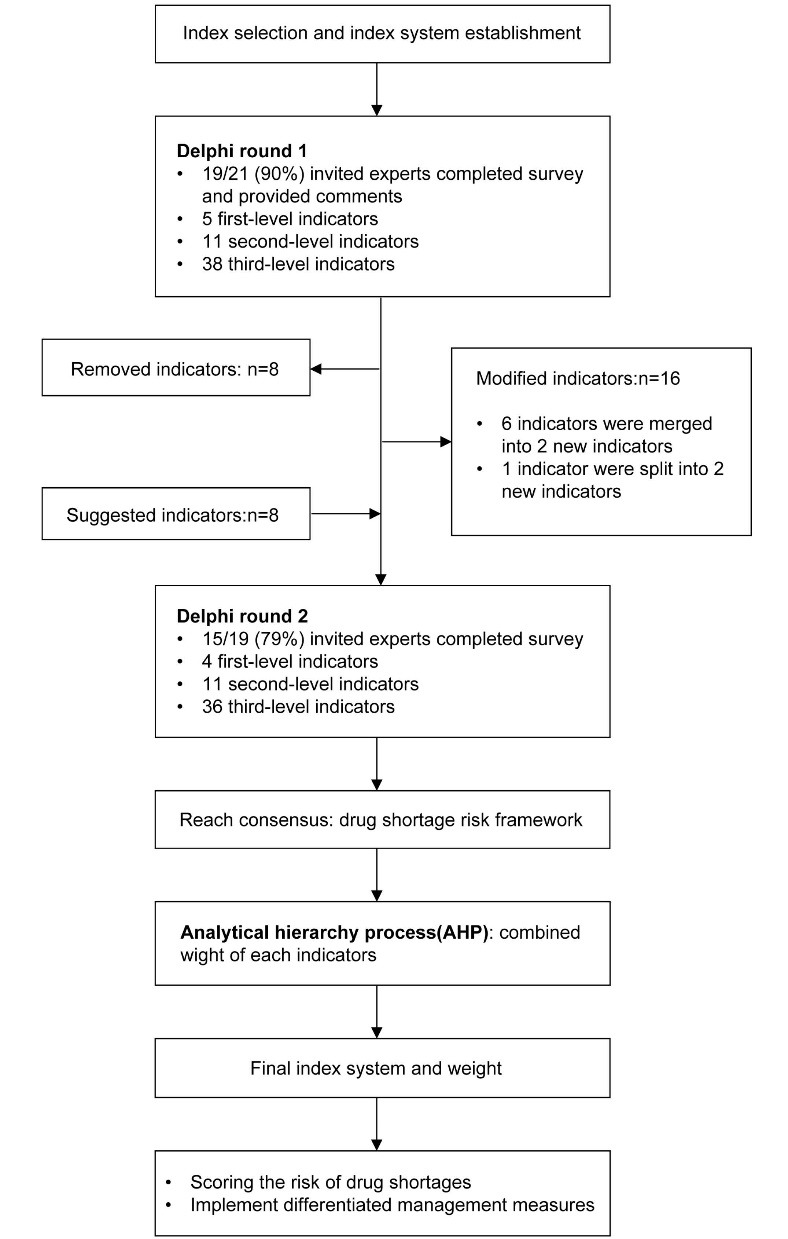
Flow Diagram of the Study Process. Abbreviation: AHP, analytic hierarchy process.

 A modified Delphi method was chosen by consensus among experts and was developed through a two-round Delphi consultation. We use the AHP model to compare and rank indexes. The duration of the study was 10 months. Index selection was started in May 2020, two-round of Delphi consultation were completed in July 2020, indicator system construction was completed in October 2020, and comprehensive evaluation of shortage drugs in 2020 was completed in February 2021. A study flow diagram is shown in Figure 1.

###  Construct the Initial Assessment Indexes

 According to the modified Delphi method, we assembled a research group including three experts specializing in the fields of pharmacy and public health and responsible for developing the initial indexes of evaluation of drug shortage risk. The initial indexes were developed based on literature,^5,22^ documents issued by the State Council,^23^ the China National Health Commission,^13,14^ and research group opinion.

###  Inclusion Criteria of Experts

 To make the group decision more reliable, 21 experts from five provinces or municipalities in China were selected for the Delphi survey. No data related to the medical institution of the expert is involved in this study, the consent of the medical institution is not required. The inclusion criteria were as follows: (1) more than 10 years of working experience in a medical institution (clinical pharmacy, pharmacy administration, or clinical medicine) or distribution enterprise (drug management), (2) deputy senior or above the position of professional or business administrator, and (3) voluntary participation in this survey and sufficient time and energy to complete the entire process.

###  Delphi Process

 We conducted a two-round Delphi survey following the guidelines of Conducting and Reporting Delphi Studies.^24^ A group of experts arrived at a consensus after two rounds of the Delphi process. Each round of the questionnaire was delivered and received by email, and respondents were asked to complete questionnaires within two weeks. Experts who did not complete a round of the survey were not invited to subsequent rounds. Experts were required to rate the rationality and importance of the indicators in each round with ‘agree,’ ‘modified,’ or ‘disagree.’ Experts were allowed to provide revision comments for each index. Importance was rated between 1 and 5 using a 5-point Likert scale: a score of 5 means extremely important, 4 means very important, 3 means important, 2 means generally important, and 1 means unimportant. Expert’s judgment criteria (*Ca*) and degree of familiarity with each indicator (*Cs*) were subsequently rated. The judgment criteria were based on four aspects: theoretical analysis, practical experience, reference literature at home and abroad, and intuitive feeling (see Table S1, Supplementary file 1). Experts’ familiarity with the index was categorized as follows: extremely familiar (1.00 point), very familiar (0.80 points), generally familiar (0.60 points), less familiar (0.40 points), and unfamiliar (0.20 points).

###  Inclusion Criteria Of Indexes

 The coefficient of variation (*CV*) and the average value of the importance score were used to determine the index. The inclusion criteria of indexes based on the critical value method^25^ were as follows: average value of the importance score ≥3.34 and *CV* ≤0.31 in the first round, and average value of the importance score ≥3.47 and *CV *≤0.20 in the second round. Other indicators whose mean value or CV did not meet the inclusion criteria were adjusted or deleted based on expert opinion. The first-round questionnaire results were analyzed and fed back to respondents before the second round of consultation. Indexes not gaining consensus in the first round were repeated in the subsequent survey until consensus was reached and the index system was constructed.

###  Weight Determination of Each Indicator

 The weight of each final indicator was rated by experts using the AHP after index determination.Hierarchical models including three levels were built using AHP to construct complex problems.^26^ Then, a judgment matrix for pairwise comparisons was created using Satty’s fundamental scales of 1-9^27^ (see Table S2, Supplementary file 1) to evaluate the relative importance of each index at the same hierarchical level. We calculated the initial weight and combination weight of each indicator and conducted consistency testing using yaahp12.4 (yaahp software, Taiyuan, Shanxi, China).

###  Statistical Analysis

 All data were entered in Microsoft Excel 2013 and checked twice. SPSS 24 software was used for statistical analysis. Descriptive statistics were expressed as frequency, percentage, rate, mean, and standard deviation. The formula used for authority coefficient (*Cr*) calculation is: *Cr* = (*Ca+Cs*)/2, with *Ca *representing experts’ judgment criteria and *Cs* representing the degree of experts’ familiarity with each indicator. The degree of coordination of experts’ opinions is expressed by Kendall’s coefficients of concordance (Kendall’s *W)* and chi-square test, and the average random consistency index was used to test the satisfactory consistency of different judgment matrices using yaahp 12.4 (yaahp software, Taiyuan, Shanxi, China). Consistency ratio (*CR*)≤0.10 indicated that the judgment matrix had satisfactory consistency.^28^ When *CR* >0.10, the judgment matrix required adjustment until *CR* ≤0.10.^28^

###  Comprehensive Evaluation

 We designed a rating scale of the drug shortage risk by converting the weight value of each three-level index into a score on a 100-point scale (see Table S3, Supplementary file 1). Data on drug shortages reported by medical institutions through the ‘Drug Classification and Procurement System of Hunan Province’^29^ from January 1 to December 31, 2020 were collected. A total of 203 medical institutions in the province participated in the reporting process as monitoring posts. Based on the above data, we carried out a risk assessment of drug shortages in Hunan province. The risk score for each drug was calculated as the sum of the scores of each indicator. Total scores ranged from 0 to 100, with higher values indicating a higher risk of drug shortage.

## Results

###  Characteristics of Experts

 We invited 21 experts to participate in the Delphi process by email. In round one, 21 questionnaires were distributed with a recovery rate of 90% (19/21), while 79% (15/19) completed and returned the questionnaires in round two. Participants in the consultation had an average age of 47 years and were from five provinces or municipalities, including Hunan, Guangdong, Yunnan, Jiangsu, and Chongqing in China. Details of the demographic characteristics of the experts are summarized in Table S4 in Supplementary file 1.

###  Authority Coefficient of Experts

 We calculated the authority coefficient of the experts based on self-evaluation scores. In the first round, the average authority coefficient of 19 experts was 0.88, increasing to 0.90 in the second round. In general, an authority coefficient of ≥0.70 indicates ‘highly credible.’^30^

###  Degree of Coordination Among Experts

 We evaluated the degree of coordination among experts with Kendall’s* W*. Generally, Kendall’s *W* is between 0 and 1; the greater the value, the higher the coordination degree of experts. In the first round of the Delphi process, Kendall’s *W* was 0.44 (χ^2^ = 441.16, *P*= .00). In the second round, Kendall’s *W* was 0.49 (χ^2^ = 370.44, *P*= .00). This indicated that the degree of coordination among experts in the second round was improved compared with the first round, and the degree of coordination among experts in both rounds was good.

###  Establishment of the Index System

 The initial index framework, including five first-level, 11 second-level, and 38 third-level indexes, was developed based on literature, government documents, and research group opinion (see Table S5, Supplementary file 1).^5,7,14,22,31^ Only indicators that met the inclusion criteria were included in the final index system. After the two-round Delphi process, eight third-level indicators were deleted. Four first-level indexes, five second-level indexes, and seven third-level indexes were modified. Eight new indexes were added, namely ‘Special classification’ (B2), ‘Emergency drugs’ (C3), ‘Detoxification drugs’ (C4), ‘Drugs for rare diseases’ (C5), ‘Other drugs’ (C6), ‘Number of manufacturers in province’ (B7), ‘Manufactured solely’ (C17), and ‘Number of manufacturers ≥2’ (C18). The final index system included four first-level indexes, 11 second-level indexes, and 36 third-level indexes (see Table 1).

**Table 1:** Final Index System for Evaluating the Risk of Drug Shortages in China

**First-Level Indexes (Code)**	**Second-Level Indexes (Code)**	**Third-Level Indexes (Code)**
(A1) Pharmaceutical properties	(B1) Essential drug classification	(C1) Essential drugs (C2) Nonessential drugs
	(B2) Special classification	(C3) Emergency drugs (C4) Detoxification drugs (C5) Drugs for rare diseases (C6) Other drugs
	(B3) Availability or alternatives	(C7) Alternative exists (C8) Full alternative does not exist^a^ (C9) No alternative
	(B4) Clinically necessary	(C10) Diagnose and treat diseases that are life-threatening or seriously impair quality of life (C11) Life-sustaining, cure disease or delay progression of disease significantly, including the diagnosis of these diseases (C12) Discontinuity of treatment has a significant impact on clinical diagnosis, treatment, and health outcomes of patient
(A2) Supply stability	(B5) Duration of short supply^b^	(C13) Time of short supply ≥6 months (C14) Time of short supply ≥3 months (C15) Time of short supply ≥1 month
	(B6) Scope of short supply	(C16) Cities with short supply ≤5 (C17) Cities with short supply >5
	(B7) Number of manufacturers in province	(C18) Manufactured solely (C19) Number of manufacturers ≥2
(A3) Drug accessibility	(B8) Number of medical institutions or distribution enterprises experiencing drug shortages	(C20) Number of medical institutions or distribution enterprises experiencing short supply ≤5 (C21) Number of medical institutions or distribution enterprises experiencing short supply between 6 and 10 (C22) Number of medical institutions or distribution enterprises experiencing short supply >10
	(B9) Categories of medical institutions experiencing drug shortages	(C23) All are primary medical institutions (C24) All are secondary medical institutions (C25) All are tertiary medical institutions (C26) Primary and secondary/tertiary medical institutions (C27) Secondary and tertiary medical institutions (C28) Primary, secondary, and tertiary medical institutions
(A4) Causes of shortage	(B10) Supply related causes	(C29) Geographical remoteness (C30) Renovation of production line (C31) Shortage of raw materials (C32) Monopoly of raw materials
	(B11) Demand related causes	(C33) Trading with low price (C34) Low clinical demand (C35) Failure of bid or bid rejection (C36) Limit order

^a^ There are significant differences in clinical application, diagnosis, treatment effect, and special population medication between drugs in short supply and the alternative option due to dosage form, specification, or route of administration. ^b^ We considered the time of short supply to be ≥6 months if the supplier is unable to anticipate a resumption of supply.

###  Weight Determination of Each Indicator

 The normalized weight and combination weight of each final indicator were calculated using AHP (see Table 2). A higher weight indicated that the indicator was more important for risk assessment. The *CR* of each modified judgment matrix was lower than 0.10, indicating that all of the judgment matrices had good consistency (see Table S6, Supplementary file 2). For first-level indicators, ‘supply stability’ (A2), ‘causes of shortage’ (A4), ‘medicine availability’ (A3), and ‘pharmaceutical properties’ (A1), the weight values were 0.3253, 0.2489, 0.2398, and 0.1860, respectively.

**Table 2:** The Weight Values of Three-Level Index System

**Index Type**	**Code of Indexes**	**Weight**	**Combined Weight**
First-level indexes	A1	0.1860	0.1860
	A2	0.3253	0.3253
	A3	0.2398	0.2398
	A4	0.2489	0.2489
Second-level indexes	B1	0.0913	0.0170
	B2	0.0575	0.0107
	B3	0.4825	0.0898
	B4	0.3686	0.0686
	B5	0.4242	0.1380
	B6	0.3403	0.1107
	B7	0.2355	0.0766
	B8	0.5413	0.1298
	B9	0.4587	0.1100
	B10	0.6071	0.1511
	B11	0.3929	0.0978
Third-level indexes	C1	0.6647	0.0113
	C2	0.3294	0.0056
	C3	0.4393	0.0047
	C4	0.2804	0.0030
	C5	0.1963	0.0021
	C6	0.0841	0.0009
	C7	0.0668	0.0060
	C8	0.1938	0.0174
	C9	0.7405	0.0665
	C10	0.5627	0.0386
	C11	0.2143	0.0147
	C12	0.2216	0.0152
	C13	0.6514	0.0899
	C14	0.2413	0.0333
	C15	0.1072	0.0148
	C16	0.2276	0.0252
	C17	0.7733	0.0856
	C18	0.6867	0.0526
	C19	0.3133	0.0240
	C20	0.1032	0.0134
	C21	0.2727	0.0354
	C22	0.6240	0.0810
	C23	0.0300	0.0033
	C24	0.0573	0.0063
	C25	0.1755	0.0193
	C26	0.1000	0.0110
	C27	0.2173	0.0239
	C28	0.4209	0.0463
	C29	0.0609	0.0092
	C30	0.1112	0.0168
	C31	0.3508	0.0530
	C32	0.4772	0.0721
	C33	0.2219	0.0217
	C34	0.2577	0.0252
	C35	0.2924	0.0286
	C36	0.2280	0.0223

###  Evaluation of the Risk of Drug Shortage

 A total of 370 drugs (453 dosage strengths) reported shortages from January 1 to December 31, 2020 in Hunan province. We excluded 67 drugs (70 dosage strengths) with a short supply time of less than one month and calculated the total risk scores of each remaining drug (383 dosage strengths). Based on the risk score, all drugs in shortage were classified as high risk (70-100 points), medium risk (40-69 points), or low risk (0-39 points). Among them, six drugs (dosage strength) were at high risk: sodium thiosulfate (0.64 g), posterior pituitary lobe hormones (1 mL:6 IU), protamine sulfate (5 mL:50 mg), thrombin (500 U), urokinase (10 WU), and rotundine sulfate (2 mL:60 mg) (see Table 3).

**Table 3:** Evaluation Scores of High-Risk Drugs

**Generic ** **Name**	**Dosage Form**	**Dosage Strength**	**Scores of Each Rated Item**^a^											**Total Evaluation Scores**
			**Item 1**	**Item 2**	**Item 3**	**Item 4**	**Item 5**	**Item 6**	**Item 7**	**Item 8**	**Item 9**	**Item 10**	**Item 11**	
Thiosulfate	Injection	0.64 g	1.56	0.65	9.17	2.10	12.39	11.80	7.25	11.17	6.38	7.31	13.47	83.25
Posterior pituitary lobe hormones	Injection	1 mL:6 UI	1.56	0.65	0.83	5.32	12.39	11.80	3.31	11.17	6.38	17.25	10.40	81.06
Protamine sulfate	Injection	5 mL:50 mg	1.56	0.65	9.17	2.10	12.39	11.80	3.31	11.17	3.29	17.25	6.46	79.15
Thrombin	Freeze-dried powder injection	500 U	1.56	0.12	0.83	2.10	12.39	11.80	3.31	11.17	3.29	19.57	10.40	76.54
Urokinase	Injection	10 WU	0.77	0.12	0.83	2.10	12.39	11.80	3.31	11.17	3.29	17.25	10.00	73.03
Rotundine sulfate	Injection	2 mL:60 mg	0.77	0.12	0.83	2.10	12.39	11.80	3.31	11.17	6.38	17.25	6.06	72.18

^a^Item 1: Essential drugs classification, Item 2: Special classification, Item 3: Availability of Alternati­ves, Item 4: Clinically necessary, Item 5: Duration of short supply, Item 6: Scope of short supply, Item 7: Number of manufacturers in province, Item 8: Number of medical institutions experiencing drug shortages, Item 9: Categories of medical institutions experiencing drug shortages, Item 10: Supply-related causes, Item 11: Demand-related causes.

 The characteristics of the drugs at different risk levels are shown in Table S7 in Supplementary file 2. Most drugs are at low shortage risk, and more than 90% of drug shortages were local (cities with short supply ≤5) and had alternatives. Only 3.66% of drugs with more than 10 medical institutions experienced short supply. Two findings were worth noting. First, more than half of the drugs in shortage (54.31%) were essential drugs. In China, drugs are deemed essentially when they are clinically necessary, safe, effective, and reasonable in price. As such, medical institutions should prioritize the use of essential drugs and focus on ensuring their supply. Second, half of the shortage (50.43%) was reported by primary medical institutions. This indicates that the shortage in primary medical institutions is more serious than that in secondary and tertiary hospitals.

**Figure 2:**
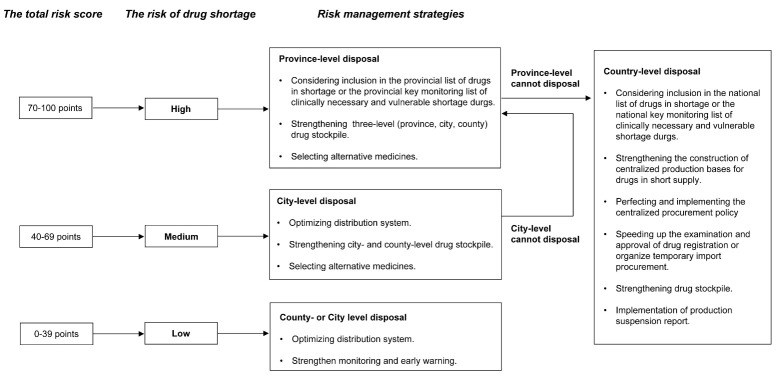
The Graded Risk-Management Strategies for Drugs at Different Risk Levels.

 Based on the risk assessment score, we built a decision tree for risk management strategies according to the features of drugs at different risk levels (see Figure 2). The risk assessment and graded management model has been approved by the Health Commission of Hunan Province for the monitoring, early warning, and selection process of drugs in shortage.

## Discussion

 Drug shortages have become a global phenomenon. How to scientifically and effectively identify drugs experiencing genuine shortages is critical for solving this problem. Using the modified Delphi method combined with AHP, we constructed an index system with universal applicability and practicability for the risk assessment of drug shortages and applied the system to guide the graded response to drug shortage and differential management strategies to address these shortages.

 The Delphi method can avoid the interplay between of experts’ mindset and the bias of ‘following the opinion of leaders,’ ensuring that the outcome is more objective and accurate than that of traditional group discussion.^32,33^ The AHP objectifies subjective judgment by proposing a quantitative scale and enlarging the weight gap between indicators to refine the identification of key risk indicators.^26^ The AHP is especially suitable for complex problems such as drug shortage risk assessments which are difficult to analyze with quantitative methods.^34,35^ The Delphi method conducted before the AHP screens out the key risk indicators from a wide range of potential indexes and reduces the difficulty of statistical calculations that might be needed due to an excessive number of variables.^26^

 In this study, a three-level index system with four first-level indicators, 11 second-level indicators, and 36 third-level indicators was developed using a two-round Delphi process. The weight value of an indicator reflects its importance.^36^ Of all first-level indicators, ‘supply stability’ (A2) was the most important. The most influential second-level indexes were ‘duration of short supply’ (B5) and ‘scope of short supply’ (B6). This suggests that the stability of drug supply is one of the most important factors for identifying and measuring the risk of drug shortage. ‘Causes of shortage’ (A4) was almost of equal importance to ‘drug accessibility’ (A3). In the above two first-level indicators, the highest scores are associated with ‘supply related causes’ (B10) and ‘number of medical institutions or distribution enterprises experiencing drug shortages’ (B8), respectively. This indicated that (1) it is necessary to understand the situation of production and raw material purchasing of drug manufacturers in a timely manner, and (2) adequate attention should be paid to the simultaneous reporting of shortages by multiple medical institutions or distribution enterprises. For the first-level indicator ‘pharmaceutical properties’ (A1), the most important second-level indicator subordinate was ‘alternatives availability’ (A2). This means that the availability of alternative medicines is important to address shortages. For drugs that can be substituted, experts should guide the selection of alternatives and assess the health risk of therapeutic substitution to ensure safe and rational drug use.^13^ For drugs with no alternatives, several measures such as strengthening drug stockpiles and optimizing distribution systems can be taken to ensure supply.

 Based on the results of the risk evaluation of drug shortages, half of the shortage was reported by primary medical institutions. This may be attributed to the unsatisfactory implementation of the “hierarchical medical system” and the imperfect of “separation of dispensing from prescription (SDP)” in China to a certain extent. Despite the government’s advocacy of a “hierarchical medical system” in recent years, the majority of patients in China still prefer tertiary medical institutions due to the gap in the size, equipment, and treatment level of medical institutions at all levels. In China, although primary medical institutions account for the largest proportion among all types of medical institutions (94.61%), the proportion of patients attending primary healthcare institutions is not high (53%).^37^ China has not yet realized the SDP,^38^ and the gap between treatment level and patient resources leads to the passive and weak position of primary medical institutions in ensuring drug supply. The shortage of drugs in primary medical institutions is more prominent due to the lack of patient resources, basic drug demand, and the fact that most primary medical institutions are located in less-developed regions, and distribution enterprises are reluctant to deliver due to relatively low-profit margin. Primary medical institutions play an essential role in the treatment of common diseases, especially for patients in rural or remote areas. More efforts should be made to ensure drug supply in primary medical institutions.

 Up to now, no other countries are reporting the characteristics of drug shortages at different levels of medical institutions. There are great differences between China and most developed countries in the classification of medical institutions, the scale and functional positioning of hospital pharmacies and so on. In most developed countries, such as the United States, the United Kingdom, Germany, and France, have achieved the SDP, hospital pharmacies mainly for inpatients.^38^ In the United States, developed retail pharmacy networks account for 70% of drug sales, while medical institutions account for less than a third.^38^ Patients usually go to retail pharmacies to obtained medicines. In China, medical institutions account for 85% of drug sales.^38^ Due to long-standing buying habits, differences in drug types and prices between hospitals and retail pharmacies, relatively good drug reputation in hospitals, and restrictions on prescription outflow in hospitals, patients generally choose to get drugs in hospital pharmacies by prescription. Once there is a drug shortage in medical institutions, the treatment of patients will be seriously affected. Based on the above, although the shortages in medical institutions among different countries may not be comparable, we expect that the findings of our study could provide a certain degree of reference for other countries to alleviate and solve the problem of drug shortage.

 Based on the risk assessment score, we built a graded risk-management strategy decision tree (see Figure 2) and applied it to the monitoring, early warning, and selection process of drugs in shortage in Hunan province. Drugs with high risk are assigned to the provincial level through a series of measures. If the intervention of relevant provincial departments has little effect, the case is assigned to the state. For medium-risk drugs, shortages are managed by municipal monitoring and warning centers, and if not resolved at the city level, are reported to the province or further to the state. Low-risk drugs are handled at the county or city level, with continuous monitoring as the main measure. The three-level framework (province, city, county) management effectively filters invalid information layer by layer and increases management efficiency. Classification management by risk level further saves limited public health resources and is more beneficial for drugs in genuine short supply.

 Several studies have assessed the risk of drug shortages.^5,22,39^ A study in Italy^5^ constructed an algorithm to measure the impact of shortage or unavailability on public health in Europe by using the three-dimension indexes of ‘type of disease,’ ‘therapeutic alternatives,’ and ‘market shares of product.’ However, the score weight criteria of each indicator were derived from expert consensus. Shang et al^22^ established a risk evaluation index system for shortages of essential drugs in China. However, the index system was only applicable to essential drugs and could not completely meet the evaluation needs of non-essential drugs. A study by Huang et al^39^ constructed a selection index system for a drug shortage list based on AHP. This index system is only applicable to the selection of a drug shortage list and requires data from all links in the pharmaceutical supply chain, including production, circulation, and use. However, data accessibility is not mentioned in the study and practical application was not conducted.

 Our study has several limitations. First, in the Delphi process, considering the feasibility, objectivity, funding, and time limit of the study, we included only experts in medical institutions and distribution enterprises, which may have led to the exclusion of comments from experts in other stakeholder group representatives such as legislative/regulatory departments, pharmaceutical manufacturer and ingredients supplier and so on. Although personnel from legislative/regulatory departments are not included in this study, the initial index framework was developed mainly based on government documents formulated by the State Council and the National Health Commission, which can largely reflect the opinions of legislative/regulatory. Due to relevant policy support, it is profitable for the pharmaceutical manufacturer or ingredients supplier to make a drug in shortage. The inclusion of them in the expert group may affect the objectivity of the assessment indicators of drug shortage risk. Second, the weight value of each index depends on the subjective judgment of experts and may not reflect the true preferences of policy-makers and decision-makers. Whenever participants were involved in the AHP stage, the assessment process had to be re-performed and the results will change accordingly. All the experts in this study were strictly selected and required to complete Delphi questionnaires carefully, accurately, and timely to ensure the quality of the study. Third, it was challenging to develop a set of indicators that apply to all regions in China. The index system has certain requirements for data acquisition, and not every province has access to all information needed for risk assessment.

## Conclusions

 A hierarchical indexed system for the risk assessment of drug shortages in China was established for the first time, using a modified Delphi process with AHP. The evaluation scores could provide clues for guiding the graded management of drugs at different risk levels. Decision trees for risk-management strategies have been established for the features of drugs at different risk levels. This study provides an objective and practical tool to guide risk assessment of drug shortages and a scientific basis for the classification and management of drugs with different risk levels. However, this risk assessment index system is not set in stone, but will require constant improvement in its practical application. We expect that the index system will contribute to the alleviation and solution of drug shortages, and thereby the reduction of disease burden and the establishment of a healthy China.

## Acknowledgments

 The authors gratefully acknowledge the Health Commission of Hunan Province and the members of the Delphi panel who provided their expertise and experience: Zaixin Yu, Xiangmin Li, Jianglin Zhang, Yaoyun Tang, Yu Zhang, Jun Quan, Sheng Deng, Min Yan, Ling Tang (Xiangya Hospital Central South University), Ping Xu (The Second Xiangya Hospital of Central South University), Xiaocong Zuo (The Third Xiangya Hospital of Central South University), Xin Zhao (Hunan Children’s Hospital), Xiaoke Wen (Hunan Provincial Maternal and child Health Care Hospital), Shujia Kong (Yunnan Cancer Hospital), Can Luo (Jiangsu Province Hospital), Yan Qian (The Second Affiliated Hospital of Chongqing Medical University), Haiyan Lao (Guangdong Provincial People’s Hospital), Tao Liu (Sun Yat-Sen University Cancer Center), Qunli Yuan (China Resources Hunan Ruige Pharmaceutical Co., Ltd.) and Zehua Wang (Sinopharm Group Hunan Co., Ltd.).

## Ethical issues

 Ethical approval was not necessary for the study because this research does not involve human subjects, human material, human tissues, or human data.

## Competing interests

 Authors declare that they have no competing interests.

## Authors’ contributions

 YS wrote the draft of the manuscript and interpreted the results. YS and QP collected and analysed the data. BZ designed the study and provided administrative and material support. SS and JD was involved in editing and revising each draft and provided overall guidance. SL, TY, ZG, and ZC provided administrative support and comments and suggestions in revisions of the paper. All authors approved the final submitted version.

## Disclaimer

 The funders had no role in the study design, data collection, data analysis, data interpretation and in writing the manuscript.

## Funding

 This work was supported by the Clinical Pharmacy Research Foundation of Hunan Medical Association (grant number HMA202001001) and the Hospital Pharmacy Research Foundation of Hunan Pharmaceutical Association (grant number 2020YXH007).

## Supplementary files


Supplementary file 1 contains Tables S1-S5.

Supplementary file 2 contains Tables S6-S7.
