# Supplementary material for: Establishment and Application of an Index System for the Risk of Drug Shortages in China: Based on Delphi Method and Analytic Hierarchy Process
Source: Int J Health Policy Manag. 2022 Mar 16;11(12):2860–8. doi: 10.34172/ijhpm.2022.6360 (PMC10105199; doi:10.34172/ijhpm.2022.6360)
Supplement: Supplementary file 1 — contains Tables S1-S5. [file ijhpm-11-2860-s001.pdf]

Article title: Establishment and Application of an Index System for the Risk of Drug Shortages in China: Based on Delphi Method and Analytic Hierarchy Process.

Journal name: International Journal of Health Policy and Management (IJHPM)

Authors' information: Yin Shi[1,2,3,4], Shusen Sun[1,2,3,4,5], Jing Deng[6], Shao Liu[1,2,3,4], Tao Yin[1,2,3,4], Qilin Peng[1,2,3,4], Zhicheng Gong[1,2,3,4], Zihua Cheng[2,3,7], Boting Zhou[1,2,3,4]*

[1] Department of Pharmacy, Xiangya Hospital, Central South University, Changsha, China.

[2] National Clinical Research Center for Geriatric Disorders, Xiangya Hospital, Central South University, Changsha, China.

[3] Hunan Drug Shortage Surveillance and Early Warning Center, Changsha, China.

[4] The Hunan Institute of Pharmacy Practice and Clinical Research, Changsha, China.

[5] Department of Pharmacy Practice, College of Pharmacy and Health Sciences, Western New England University, Springfeld, MA, USA.

[6] Department of Epidemiology and Health Statistics, XiangYa School of Public Health, Central South University, Changsha, China.

7 Department of General Surgery, Xiangya Hospital, Central South University, Changsha, China.

(Corresponding author: botingzhou0918@126.com)

Supplementary file 1. Contains Table S1-Table S5

Table S1: The judgment criteria of experts

Table S2: Fundamental 9-point scale

Table S3: The rating scale for the drug shortage risk

Table S4: Demographic characteristics of experts

Table S5: The initial index system for evaluating the risk of drug shortages

Table S1 The judgment criteria of experts

Judgment basis	Degree of contribution to expert judgment		
	**large**	**medium**	**small**
Theoretical analysis	0.30	0.20	0.10
Practical experience	0.45	0.35	0.20
Reference literature at home and abroad	0.20	0.15	0.10
Intuitive feeling	0.05	0.05	0.05

Table S2 Fundamental 9-point scale

Scale values[a]	The importance of Index A relative to index B[b]
1	Equally important
2	The degree of importance between "Equally important" and "General important"
3	General important
4	The degree of importance between "General important" and "Important"
5	Important
6	The degree of importance between "Important" and "very important"
7	Very important
8	The degree of importance between "Very important" and "Extremely important"
9	Extremely important

[a] The reciprocal of the score value (1-1/9) indicated an inversion of the importance of index A relative to index B. (eg. A scale value of 1/3 means the index B was general important relative to index A.)

[b] 'A' represent the row indicator in the judgment matrix. "B" represent the column indicators in the judgment matrix.

Table S3 The rating scale for the drug shortage risk

Rated items (n=11)	Options[a]	Scores
**Pharmaceutical properties**		
(1)Essential drug classification	Essential drugs	1.56
	Nonessential drugs	0.77
(2)Special classification	Emergency drugs	0.65
	Detoxification drugs	0.41
	Drugs for rare diseases	0.29
	Other drugs	0.12
(3)Availability or alternatives	Alternative exists	0.83
	Full alternative does not exist[b]	2.40
	No alternatives	9.17
(4)Clinically necessary	Diagnose and treat diseases that are life-threatening or seriously impaired quality of life	5.32
	Life-sustaining, cure disease or delay progression of the disease significantly, including the diagnosis of these diseases	2.03
	Discontinuity of treatment has a significant impact on clinical diagnosis and treatment and the health outcomes of patients	2.10
**Supply stability**		
(5)Duration of short supply[c]	Time of short supply ≥6 months	12.39
	Time of short supply ≥3 months	4.59
	Time of short supply ≥1 months	2.04
(6)Scope of short supply	Cities with short supply ≤5	3.47
	Cities with short supply >5	11.80
(7)Number of manufacturers in province	Manufactured solely	7.25
	Number of manufacturers ≥2	3.31
**Drug accessibility**		
(8)Number of medical	Medical institutions / distribution enterprises	1.85

institutions or distribution enterprises experiencing drug shortages	experiencing short supply ≤5	
	Medical institutions / distribution enterprises experiencing short supply between 6-10	4.88
	Medical institutions / distribution enterprises experiencing short supply >10	11.17
(9)Categories of medical institutions experiencing drug shortages	All are primary health care institutions	0.45
	All are secondary health care institutions	0.87
	All are tertiary health care institutions	2.66
	Primary and secondary health care institutions	1.52
	Secondary and tertiary health care institutions	3.29
	Primary, secondary, and tertiary health care institutions	6.38
**Causes of shortage**		
(10)Supply related causes*	Geographical remoteness	1.27
	Renovation of production line	2.32
	Shortage of raw materials	7.31
	Monopoly of raw materials	9.94
(11)Demand related causes*	Trading with low price	2.99
	Low clinical demand	3.47
	Failure of bid or bid rejection	3.94
	Limit order	3.07

The total evaluation score of drug shortage risk was obtained by summing up the points of 11 rated items.

[a] Items marked with an asterisk(*) can be multiple choices, and the remaining items are single choices.

[b] There are significant differences in clinical application, diagnosis, treatment effect, and special population medication between drugs in short supply and the alternative option due to dosage form, specification, or route of administration.

[c] We considered the time of short supply to be ≥6 months if the supplier is unable to anticipate a resumption of supply.

Table S4 Demographic characteristics of experts

Characteristics	Mean (SD) or n (%)	
	**Round I (n=19)**	**Round 2 (n=15)**
**Age (years)**	47.47 (5.36)	47.33 (5.91)
**Gender**		
Male	8 (42.11)	5 (33.33)
Female	11 (57.89)	10 (66.67)
**Highest degree**		
Doctor	10 (52.63)	8 (53.33)
master	6 (31.58)	6 (40)
Undergraduate	3 (15.79)	1(6.67)
**Professional title**		
Senior professional title	12 (63.16)	10 (66.67)
Deputy senior professional title	5 (26.32)	5 (33.33)
Company executive	2 (10.53)	0
**Position**		
Director/deputy director	15 (78.95)	13 (86.67)
Business administrator	2 (10.53)	0
other	2 (10.53)	2 (13.33)
**Field of expertise**		
Clinical pharmacy	13 (68.42)	13 (86.67)
Clinical medicine	4 (21.05)	2 (13.33)
Other	2 (10.53)	0
**Work experience (years)**		
10-19	8 (42.11)	7 (46.67)
20-29	7 (36.84)	5 (33.33)
30-39	4 (21.05)	3 (20)
**Provinces or city**		
Hunan	14 (73.68)	10 (66.67)
Guangdong	2 (10.53)	2 (13.33)
Yunan	1 (5.26)	1 (6.67)

Jiangsu	1 (5.26)	1 (6.67)
Chongqing	1 (5.26)	1 (6.67)

Table S5 The initial index system for evaluating the risk of drug shortages

First-level indicators	Second-level indicators	Third-level indicators
(A1) Pharmaceutical properties	(B1) Clinically necessary	(C1) Diagnose and treat diseases that are life-threatening or seriously impaired quality of life
		(C2) Life-sustaining, cure disease or delay progression of the disease significantly, including the diagnosis of these diseases
		(C3) Discontinuity of treatment has a significant impact on clinical diagnosis, treatment and the health outcomes of patient
	(B2) Drug category	(C4) Essential drugs
		(C5) Nonessential drugs
(A2) Availability or alternatives	(B3) Alternative exists	(C6) Drugs with similar clinical diagnosis and treatment effects
		(C7) Drugs with the same classification of pharmacological effects
		(C8) Drugs with the same chemical composition
		(C9) Drugs with the same generic name but the different manufacturer
	(B4) Full alternative does not exist[a]	(C10) There are significant differences in clinical application, diagnosis and treatment effect or special population medication between drug in short supply and the alternative option due to dosage form
		(C11) There are significant differences in clinical application, diagnosis and treatment effect or special population medication

		between drug in short supply and the alternative option due to specification
		(C12) There are significant differences in clinical application, diagnosis and treatment effect or special population medication between drug in short supply and the alternative option due to route of administration
	(B5) No alternatives	(C13) Therapeutic alternatives have the attributes listed in indicators C6-C12 are not available
(A3) Supply stability	(B6) Duration of short supply[b]	(C14) Time of short supply ≥6 months
		(C15) Time of short supply ≥3 months
		(C16) Time of short supply ≥1 months
	(B7) Scope of short supply	(C17) City with short supply only one
		(C18) Cities with short supply between 2 and 3
		(C19) Cities with short supply between 4 and 5
		(C20) Cities with short supply are more than 5
(A4) The situation of medical institutions experiencing drug shortages	(B8) Number of medical institutions experiencing drug shortages	(C21) Medical institution experiencing short supply only one
		(C22) Number of medical institutions experiencing short supply between 2 and 3
		(C23) Number of medical institutions experiencing short supply between 4 and 5
		(C24) Number of medical institutions experiencing short supply are more than 5

	(B9) Categories of medical institutions experiencing drug shortages	(C25) Primary health care institutions
		(C26) Secondary health care institutions
		(C27) Tertiary health care institutions
		(C28) Primary and secondary health care institutions
		(C29) Secondary and tertiary health care institutions
		(C30) Primary, secondary, and tertiary health care institutions
(A5) Causes of shortage	(B10) Supply related ones	(C31) Geographical remoteness
		(C32) Renovation of production line
		(C33) Shortage of raw materials
		(C34) Monopoly of raw materials
	(B11) Demand related ones	(C35) Trading with low price
		(C36) Low clinical demand
		(C37) Failure of bid or bid rejection
		(C38) Limit order

[a]There are significant differences in clinical application, diagnosis, treatment effect, and special population medication between drugs in short supply and the alternative option due to dosage form, specification, or route of administration.
[b] We considered the time of short supply to be ≥6 months if the supplier is unable to anticipate a resumption of supply.
