# Supplementary material for: Establishment and Application of an Index System for the Risk of Drug Shortages in China: Based on Delphi Method and Analytic Hierarchy Process
Source: Int J Health Policy Manag. 2022 Mar 16;11(12):2860–8. doi: 10.34172/ijhpm.2022.6360 (PMC10105199; doi:10.34172/ijhpm.2022.6360)
Supplement: Supplementary file 2 — contains Tables S6-S7. [file ijhpm-11-2860-s002.pdf]

Article title: Establishment and Application of an Index System for the Risk of Drug Shortages in China: Based on Delphi Method and Analytic Hierarchy Process.

Journal name: International Journal of Health Policy and Management (IJHPM)

Authors' information: Yin Shi[1,2,3,4], Shusen Sun[1,2,3,4,5], Jing Deng[6], Shao Liu[1,2,3,4], Tao Yin[1,2,3,4], Qilin Peng[1,2,3,4], Zhicheng Gong[1,2,3,4], Zihua Cheng[2,3,7], Boting Zhou[1,2,3,4*]

[1] Department of Pharmacy, Xiangya Hospital, Central South University, Changsha, China.

[2] National Clinical Research Center for Geriatric Disorders, Xiangya Hospital, Central South University, Changsha, China.

[3] Hunan Drug Shortage Surveillance and Early Warning Center, Changsha, China.

[4] The Hunan Institute of Pharmacy Practice and Clinical Research, Changsha, China.

[5] Department of Pharmacy Practice, College of Pharmacy and Health Sciences, Western New England University, Springfeld, MA, USA.

[6] Department of Epidemiology and Health Statistics, XiangYa School of Public Health, Central South University, Changsha, China.

[7] Department of General Surgery, Xiangya Hospital, Central South University, Changsha, China.

(Corresponding author: botingzhou0918@126.com)

Supplementary file 2. Contains Table S6-Table S7

Table S6: Judgment matrix and consistency test of the indexes

Table S7: The characteristics of drugs at different risk levels

Table S6 Judgment matrix and consistency test of the indexes

Judgment matrix of first-level indexes (A1 Pharmaceutical properties, A2 Supply stability, A3 Drug accessibility, A4 Causes of shortage**)**

Consistency test	Expert 1	Expert 2	Expert 3	Expert 4	Expert 5	Expert 6	Expert 7	Expert 8	Expert 9	Expert 10	Expert 11	Expert 12	Expert 13	Expert 14	Expert 15
CR	0.0909	0.0806	0.0039	0.0618	0.0790	0.0676	0.0227	0.0891	0.0771	0.0806	0.0866	0.0989	0.0675	0.0413	0.0039
λmax	4.2428	4.2153	4.0104	4.1649	4.2109	4.1806	4.0606	4.2379	4.2059	4.2153	4.2314	4.2640	4.1801	4.1102	4.0104

Judgment matrix of second-level indexes (B1 Essential drug classification, B2 Special classification, B3Availability or alternatives, B4 Clinically necessary**)**

Consistency test	Expert 1	Expert 2	Expert 3	Expert 4	Expert 5	Expert 6	Expert 7	Expert 8	Expert 9	Expert 10	Expert 11	Expert 12	Expert 13	Expert 14	Expert 15
CR	0.0287	0.0769	0.0813	0.0023	0.0790	0.0227	0.0880	0.0822	0.0940	0.0789	0.0328	0.0000	0.0909	0.0429	0.0766
λmax	4.0766	4.2053	4.2171	4.0062	4.2109	4.0606	4.2350	4.2194	4.2510	4.2106	4.0876	4.0000	4.2428	4.1145	4.2046

Judgment matrix of second-level indexes (B5 Duration of short supply, B6 Scope of short supply, B7 Number of manufacturers in province**)**

Consistency test	Expert 1	Expert 2	Expert 3	Expert 4	Expert 5	Expert 6	Expert 7	Expert 8	Expert 9	Expert 10	Expert 11	Expert 12	Expert 13	Expert 14	Expert 15
CR	0.0053	0.0825	0.0000	0.0088	0.0961	0.0000	0.0000	0.0904	0.0904	0.0624	0.0370	0.0279	0.0176	0.0961	0.0000
λmax	3.0055	3.0858	3.0000	3.0092	3.0999	3.0000	3.0000	3.0940	3.0940	3.0649	3.0385	3.0291	3.0183	3.0999	3.0000

Judgment matrix of second-level indexes (B8 Number of medical institutions or distribution enterprises experiencing drug shortages, B9 Categories of medical institutions experiencing drug shortages**)**

Consistency test	Expert 1	Expert 2	Expert 3	Expert 4	Expert 5	Expert 6	Expert 7	Expert 8	Expert 9	Expert 10	Expert 11	Expert 12	Expert 13	Expert 14	Expert 15
CR	0.0000	0.0825	0.0000	0.0000	0.0000	0.0000	0.0000	0.0000	0.0000	0.0000	0.0000	0.0000	0.0000	0.0000	0.0000
λmax	2.0000	3.0858	2.0000	2.0000	2.0000	2.0000	2.0000	2.0000	2.0000	2.0000	2.0000	2.0000	2.0000	2.0000	2.0000

Judgment matrix of second-level indexes (B10 Supply related causes, B11 Demand related causes**)**

Consistency test	Expert 1	Expert 2	Expert 3	Expert 4	Expert 5	Expert 6	Expert 7	Expert 8	Expert 9	Expert 10	Expert 11	Expert 12	Expert 13	Expert 14	Expert 15
CR	0.0000	0.0000	0.0000	0.0000	0.0000	0.0000	0.0000	0.0000	0.0000	0.0000	0.0000	0.0000	0.0000	0.0000	0.0000
λmax	2.0000	2.0000	2.0000	2.0000	2.0000	2.0000	2.0000	2.0000	2.0000	2.0000	2.0000	2.0000	2.0000	2.0000	2.0000

Judgment matrix of third-level indexes (C1 Essential drugs, C2 Nonessential drugs**)**

Consistency test	Expert 1	Expert 2	Expert 3	Expert 4	Expert 5	Expert 6	Expert 7	Expert 8	Expert 9	Expert 10	Expert 11	Expert 12	Expert 13	Expert 14	Expert 15
CR	0.0000	0.0000	0.0000	0.0000	0.0000	0.0000	0.0000	0.0000	0.0000	0.0000	0.0000	0.0000	0.0000	0.0000	0.0000
λmax	2.0000	2.0000	2.0000	2.0000	2.0000	2.0000	2.0000	2.0000	2.0000	2.0000	2.0000	2.0000	2.0000	2.0000	2.0000

Judgment matrix of third-level indexes (C3 Emergency drugs, C4 Detoxification drugs, C5 Drugs for rare diseases, C6 Other drugs**)**

Consistency test	Expert 1	Expert 2	Expert 3	Expert 4	Expert 5	Expert 6	Expert 7	Expert 8	Expert 9	Expert 10	Expert 11	Expert 12	Expert 13	Expert 14	Expert 15
CR	0.0204	0.0963	0.0123	0.0312	0.0826	0.0227	0.0123	0.0940	0.0163	0.0212	0.0888	0.0397	0.0822	0.0339	0.0123
λmax	4.0544	4.2572	4.0328	4.0833	4.2206	4.0606	4.0328	4.2509	4.0435	4.0566	4.2371	4.1061	4.2194	4.0905	4.0328

Judgment matrix of third-level indexes (C7 Alternative exists, C8 Full alternative does not exist, C9 No alternative**)**

Consistency test	Expert 1	Expert 2	Expert 3	Expert 4	Expert 5	Expert 6	Expert 7	Expert 8	Expert 9	Expert 10	Expert 11	Expert 12	Expert 13	Expert 14	Expert 15
CR	0.0772	0.0516	0.0904	0.0685	0.0707	0.0685	0.0904	0.0707	0.0772	0.0311	0.0904	0.0772	0.0707	0.0685	0.0685
λmax	3.0803	3.0536	3.0940	3.0713	3.0735	3.0713	3.0940	3.0735	3.0803	3.0324	3.0940	3.0803	3.0735	3.0713	3.0713

Judgment matrix of third-level indexes (C10 Diagnose and treat diseases that are life-threatening or seriously impair quality of life, C11 Life-sustaining, cure disease or delay progression of the disease significantly, including the diagnosis of these diseases, C12 Discontinuity of treatment has a significant impact on clinical diagnosis, treatment and health outcomes of patient**)**

Consistency test	Expert 1	Expert 2	Expert 3	Expert 4	Expert 5	Expert 6	Expert 7	Expert 8	Expert 9	Expert 10	Expert 11	Expert 12	Expert 13	Expert 14	Expert 15
CR	0.0336	0.0904	0.0036	0.0707	0.0355	0.0176	0.0036	0.0624	0.0000	0.0516	0.0904	0.0176	0.0370	0.0516	0.0036

λmax	3.0349	3.0940	3.0037	3.0735	3.0369	3.0183	3.0037	3.0649	3.0000	3.0536	3.0940	3.0183	3.0385	3.0536	3.0037

Judgment matrix of third-level indexes (C13 Time of short supply ≥6 months, C14 Time of short supply ≥3 months, C15 Time of short supply ≥1 months**)**

Consistency test	Expert 1	Expert 2	Expert 3	Expert 4	Expert 5	Expert 6	Expert 7	Expert 8	Expert 9	Expert 10	Expert 11	Expert 12	Expert 13	Expert 14	Expert 15
CR	0.0516	0.0336	0.0176	0.0036	0.0772	0.0685	0.0370	0.0516	0.0772	0.0707	0.0088	0.0068	0.0624	0.0772	0.0176
λmax	3.0536	3.0349	3.0183	3.0037	3.0803	3.0713	3.0385	3.0536	3.0803	3.0735	3.0092	3.0070	3.0649	3.0803	3.0183

Judgment matrix of third-level indexes (C16 Cities with short supply ≤5, C17 Cities with short supply >5**)**

Consistency test	Expert 1	Expert 2	Expert 3	Expert 4	Expert 5	Expert 6	Expert 7	Expert 8	Expert 9	Expert 10	Expert 11	Expert 12	Expert 13	Expert 14	Expert 15
CR	0.0000	0.0000	0.0000	0.0000	0.0000	0.0000	0.0000	0.0000	0.0000	0.0000	0.0000	0.0000	0.0000	0.0000	0.0000
λmax	2.0000	2.0000	2.0000	2.0000	2.0000	2.0000	2.0000	2.0000	2.0000	2.0000	2.0000	2.0000	2.0000	2.0000	2.0000

Judgment matrix of third-level indexes (C18 Manufactured solely, C19 Number of manufacturers ≥2**)**

Consistency test	Expert 1	Expert 2	Expert 3	Expert 4	Expert 5	Expert 6	Expert 7	Expert 8	Expert 9	Expert 10	Expert 11	Expert 12	Expert 13	Expert 14	Expert 15
CR	0.0000	0.0000	0.0000	0.0000	0.0000	0.0000	0.0000	0.0000	0.0000	0.0000	0.0000	0.0000	0.0000	0.0000	0.0000
λmax	2.0000	2.0000	2.0000	2.0000	2.0000	2.0000	2.0000	2.0000	2.0000	2.0000	2.0000	2.0000	2.0000	2.0000	2.0000

Judgment matrix of third-level indexes (C20 Number of medical institutions or distribution enterprises experiencing short supply ≤5, C21 Number of medical institutions or distribution enterprises experiencing short supply between 6 and 10, C22 Number of medical institutions or distribution enterprises experiencing short supply >10**)**

Consistency test	Expert 1	Expert 2	Expert 3	Expert 4	Expert 5	Expert 6	Expert 7	Expert 8	Expert 9	Expert 10	Expert 11	Expert 12	Expert 13	Expert 14	Expert 15
CR	0.0624	0.0772	0.0068	0.0825	0.0772	0.0707	0.0068	0.0904	0.0176	0.0000	0.0068	0.0624	0.0624	0.0685	0.0068
λmax	3.0649	3.0803	3.0070	3.0858	3.0803	3.0735	3.0070	3.0940	3.0183	3.0000	3.0070	3.0649	3.0649	3.0713	3.0070

Judgment matrix of third-level indexes (C23 All are primary health care institutions, C24 All are secondary health care institutions, C25 All are tertiary health care institutions, C26 Primary

and secondary/tertiary health care institutions, C27 Secondary and tertiary health care institutions, C28 Primary, secondary, and tertiary health care institutions**)**

Consistency test	Expert 1	Expert 2	Expert 3	Expert 4	Expert 5	Expert 6	Expert 7	Expert 8	Expert 9	Expert 10	Expert 11	Expert 12	Expert 13	Expert 14	Expert 15
CR	0.0878	0.0969	0.0211	0.0645	0.0946	0.0979	0.0348	0.0963	0.0935	0.0723	0.0798	0.0623	0.0908	0.0513	0.0211
λmax	6.5529	6.6108	6.1332	6.4062	6.5960	6.6166	6.2193	6.6065	6.5892	6.4557	6.5030	6.3926	6.5721	6.3232	6.1332

Judgment matrix of third-level indexes (C29 Geographical remoteness, C30 Renovation of production line, C31 Shortage of raw materials, C32 Monopoly of raw materials**)**

Consistency test	Expert 1	Expert 2	Expert 3	Expert 4	Expert 5	Expert 6	Expert 7	Expert 8	Expert 9	Expert 10	Expert 11	Expert 12	Expert 13	Expert 14	Expert 15
CR	0.0992	0.0981	0.0123	0.0665	0.0790	0.0854	0.0123	0.0945	0.0895	0.0976	0.0900	0.0444	0.0822	0.0981	0.0123
λmax	4.2650	4.2620	4.0328	4.1776	4.2109	4.2280	4.0328	4.2524	4.2389	4.2606	4.2404	4.1184	4.2195	4.2620	4.0328

Judgment matrix of third-level indexes (C33 Trading with low price, C34Low clinical demand, C35 Failure of bid or bid rejection, C36Limit order**)**

Consistency test	Expert 1	Expert 2	Expert 3	Expert 4	Expert 5	Expert 6	Expert 7	Expert 8	Expert 9	Expert 10	Expert 11	Expert 12	Expert 13	Expert 14	Expert 15
CR	0.0454	0.0933	0.0000	0.0172	0.0922	0.0454	0.0000	0.0845	0.0000	0.0806	0.0806	0.0000	0.0295	0.0806	0.0000
λmax	4.1213	4.2492	4.0000	4.0458	4.2463	4.1213	4.0000	4.2257	4.0000	4.2153	4.2153	4.0000	4.0788	4.2153	4.0000

CR, consistency ratio

Table S7 The characteristics of drugs at different risk levels

	Total(n=383)	High risk[a] (n=6)	Medium risk[b] (n=39)	Low risk[c] (n=338)
**Essential drug classification**				
Essential drugs	208 (54.31%)	4 (66.67%)	30 (76.92%)	174 (51.48%)
Nonessential drugs	175 4(5.69%)	2 (33.33%)	9 (23.08%)	164 (48.52%)
**Special classification**				
Emergency drugs	38 (9.92%)	3 (50.00%)	5 (12.82%)	30 (8.88%)
Detoxification drugs	0	0	0	0
Drugs for rare diseases	0	0	0	0
Other drugs	345 (90.08%)	3 (50.00%)	34 (87.18%)	308 (91.12%)
**Availability or alternatives**				
Alternative exists	371 (96.87%)	4 (66.67%)	35 (89.74%)	332 (98.22%)
Full alternative does not exist	10 (2.61%)	0 (33.33%)	4 (10.26%)	6 (1.78%)
No alternative	2 (0.52%)	2	0	0
**Duration of short supply**				
Time of short supply ≥6 months	123 (32.11%)	6 (100.00%)	29 (74.36%)	88 (26.04%)
Time of short supply ≥3 months	91 (23.76%)	0	5 (12.82%)	86 (25.44%)
Time of short supply ≥1 months	169 (44.13%)	0	5 (12.82%)	164 (48.52%)
**Scope of short supply**				
Cities with short supply ≤5	350 (91.38%)	0	17 (43.59%)	333 (98.52%)
Cities with short supply >5	33 (8.62%)	6 (100%)	22 (56.41%)	5 (1.48%)
**Number of manufacturers in province**				
Manufactured solely	61 (15.93%)	1 (16.67%)	4 (10.26%)	56 (16.57%)
Number of manufacturers ≥2	322 (84.07%)	5 (83.33%)	35 (89.74%)	282 (83.43%)
**Number of medical institutions or distribution enterprises experiencing drug shortages**				
Medical institutions or distribution enterprises experiencing short supply ≤5	340 (88.77%)	0	9 (23.08%)	331 (97.93%)
Medical institutions or distribution enterprises experiencing short supply between 6-10	29 (7.57%)	0	22 (56.41%）	7 (2.07%)
Medical institutions or distribution enterprises experiencing short supply >10	14 (3.66%)	6 (100%)	8 (20.51%)	0

**Categories of medical institutions experiencing drug shortages**				
All are primary health care institutions	120 (34.19%)	0	0	120 (38.71%)
All are secondary health care institutions	67 (19.09%)	0	2 (5.71%)	65 (20.97%)
All are tertiary health care institutions	69 (19.66%)	0	3 (8.57%)	66 (21.29%)
Primary and secondary health care institutions	38 (10.83%)	0	7 (20%)	31 (10.00%)
Secondary and tertiary health care institutions	38 (10.83%)	3 (50%)	12 (34.29%)	23 (7.42%)
Primary, secondary, and tertiary health care institutions	19 (5.41%)	3 (50%)	11 (31.43%)	5 (1.61%)

[a] High risk represented the drug with a shortage risk score between 70-100 points.

[b] Medium risk represented the drug with a shortage risk score between 40-69 points.

[c] Low risk represented the drug with a shortage risk score between 0-39 points.
